# Learning about Time within the Spinal Cord II: Evidence that Temporal Regularity Is Encoded by a Spinal Oscillator

**DOI:** 10.3389/fnbeh.2016.00014

**Published:** 2016-02-11

**Authors:** Kuan H. Lee, Yung-Jen Huang, James W. Grau

**Affiliations:** ^1^Department of Neurobiology, Center for Pain Research, University of Pittsburgh School of MedicinePittsburgh, PA, USA; ^2^Department of Psychology, Cellular and Behavioral Neuroscience, Texas A&M UniversityCollege Station, TX, USA

**Keywords:** spinal cord, time, instrumental learning, memory, central pattern generator

## Abstract

How a stimulus impacts spinal cord function depends upon temporal relations. When intermittent noxious stimulation (shock) is applied and the interval between shock pulses is varied (unpredictable), it induces a lasting alteration that inhibits adaptive learning. If the same stimulus is applied in a temporally regular (predictable) manner, the capacity to learn is preserved and a protective/restorative effect is engaged that counters the adverse effect of variable stimulation. Sensitivity to temporal relations implies a capacity to encode time. This study explores how spinal neurons discriminate variable and fixed spaced stimulation. Communication with the brain was blocked by means of a spinal transection and adaptive capacity was tested using an instrumental learning task. In this task, subjects must learn to maintain a hind limb in a flexed position to minimize shock exposure. To evaluate the possibility that a distinct class of afferent fibers provide a sensory cue for regularity, we manipulated the temporal relation between shocks given to two dermatomes (leg and tail). Evidence for timing emerged when the stimuli were applied in a coherent manner across dermatomes, implying that a central (spinal) process detects regularity. Next, we show that fixed spaced stimulation has a restorative effect when half the physical stimuli are randomly omitted, as long as the stimuli remain in phase, suggesting that stimulus regularity is encoded by an internal oscillator Research suggests that the oscillator that drives the tempo of stepping depends upon neurons within the rostral lumbar (L1-L2) region. Disrupting communication with the L1-L2 tissue by means of a L3 transection eliminated the restorative effect of fixed spaced stimulation. Implications of the results for step training and rehabilitation after injury are discussed.

## Introduction

Neural systems provide organisms with a way to structure behavior over time and predict future events. In both cases, this requires a capacity to represent the temporal relation between events, a kind of clock (Ivry and Spencer, 2004; Mauk and Buonomano, 2004). Of course, many neural processes operate within prescribed temporal limits, and in this way, time plays a pervasive role. The concept of a clock goes beyond these temporal constraints to posit a general purpose device that has some capacity to encode a range of temporal relations. A clock of this sort must be trainable and have a lasting effect (a kind of memory). Here, we explore the possibility that spinal neurons are capable of this kind of timing.

**Figure 1:**
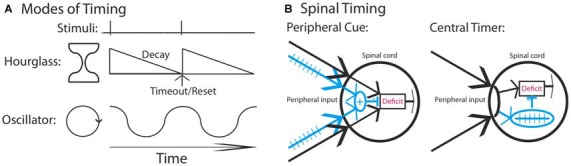
**Mechanisms that could underlie the divergent effect of regular stimulation. (A)** Stimuli that occur at a regular interval could involve a neural timer (hourglass) or an oscillator. It is assumed that a physical event initiates the process. An hourglass times over a set period, providing a marker that indicates the interval duration. An oscillator involves a cyclic device that is assumed to operate in phase with fixed spaced stimulation. **(B)** Regular stimulation could engage a distinct fiber type (blue) providing a peripheral cue that the environmental stimulus occurs at a regular temporal interval. Alternatively, a central process may abstract regularity across time and location. In both cases, once a threshold is reached, the learning deficit (red) is inhibited.

Neural models of timing have typically related timing to two types of mechanisms (Figure 1A): hourglass clocks that time over a set period based upon a chemical process that has a prescribed duration or biological oscillators that cycle rhythmically across a set period (Boulos and Terman, 1980; Karmarkar and Buonomano, 2007). Of the two, oscillators have been found to play an especially pervasive role, controlling a range of behaviors including feeding, diurnal activity, estrous cycles and seasonal rhythms (Dunlap, 1999; Bell-Pedersen et al., 2005). Prior work has shown that spinal neurons have oscillatory devices that control rhythmic behaviors, such as stepping, tail waving and scratching (for review, Duysens and Van de Crommert, 1998; Marder and Bucher, 2001; Kiehn, 2006; Hultborn and Nielsen, 2007; Guertin, 2009; Rossignol and Frigon, 2011; Frigon, 2012).

**Figure 2:**
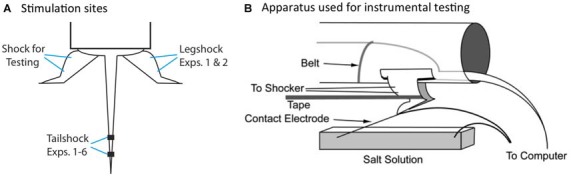
**Locus of stimulation and test apparatus. (A)** Shock stimuli are applied to the tail or leg and subjects are tested on the untreated leg. The leg used for the application of legshock and testing is counter-balanced across subjects. **(B)** The apparatus used for instrumental testing. Spinally transected rats given legshock whenever the contact electrode touches the underlying salt solution exhibit a progressive increase in flexion duration that minimizes net shock exposure. This learning is disrupted by prior exposure to variable intermittent shock.

We have previously shown that spinal neurons can detect whether an intermittent stimulus occurs at regular (predictable) or variable (unpredictable) temporal intervals (Baumbauer et al., 2008, 2009). In these studies, the lumbosacral spinal cord was isolated from the brain by means of a transection at the second thoracic (T2) vertebra. Intermittent stimulation was given by repeatedly applying a brief (80 ms) shock to one hind limb or the tail (Figure 2A). We found that 180–900 shocks given on a variable time (VT) schedule [rectangular distribution (0.2–3.8); mean: 2 s] induces an alteration in spinal function that disrupts adaptive learning (Crown et al., 2002; Baumbauer et al., 2008; for review, Grau et al., 2014). We assessed learning using an instrumental conditioning task wherein shock is applied to one hind leg whenever the leg is extended (Figure 2B). Normally, this response-shock contingency produces an increase in flexion duration that minimizes net shock exposure—a form of adaptive plasticity (Grau et al., 1998, 2012). Subjects that have previously received variable shock fail to learn when tested in this paradigm, and demonstrate a learning deficit reminiscent of learned helplessness (Maier and Seligman, 1976). Importantly, variable intermittent stimulation has: (1) a lasting effect (Crown et al., 2002); impacts learning independent of whether the stimuli are applied to the same or distant dermatomes (Joynes et al., 2003); and is not accompanied by a general inhibition of motoric behavior (Grau et al., 1998; Ferguson et al., 2006). These observations suggest that the phenomenon reflects an alteration in plastic potential, a form of metaplasticity that inhibits spinal learning and recovery (Ferguson et al., 2012; Grau et al., 2014). Interestingly, a period of instrumental training (controllable stimulation) prior to VT shock has a protective effect that blocks the induction of the learning deficit (Crown and Grau, 2001). The protective effect of controllable stimulation has been linked to the expression of brain derived neurotrophic factor (BDNF; Gómez-Pinilla et al., 2007; Huie et al., 2012b), while the adverse effect of VT stimulation has been tied to central sensitization and the cytokine tumor necrosis factor (TNF; Ferguson et al., 2006, 2012; Huie et al., 2012a). Additionally, variable intermittent nociceptive stimulation impairs recovery after a contusion injury and this effect has been linked to a down-regulation of BDNF and up-regulation of TNF (Garraway et al., 2011, 2014; Grau et al., 2004).

Electrophysiological studies have shown that the induction of the learning impairment requires stimulation at an intensity that activates unmyelinated C (pain) fibers (Baumbauer et al., 2008). In performing these studies, we made what we thought was a subtle modification to our stimulation paradigm—we began to present the intermittent shock using a fixed time (FT) schedule (2 s apart; 0.5 Hz). When 180 shocks were given, both FT and VT stimulation induced a learning impairment. However, when 900 shocks were administered, only VT stimulation produced a deficit. This implies that the presentation of an additional 720 FT shocks had a *restorative* effect that eliminated the learning impairment induced by the initial 180 shocks and reinstated the capacity for learning. Further work revealed that 720 FT shocks prevent the learning deficit induced by a 180 VT shocks and that this *protective* effect lasts 24 h (Baumbauer et al., 2009). The induction of this restorative/protective effect depends upon the NMDA receptor (NMDAR) and protein synthesis. The expression of the protective effect has been linked to BDNF (Baumbauer et al., 2009).

Interestingly, the restorative effect of FT stimulation can emerge when subjects are given two bouts of 360 FT shocks separated by 24 h (Lee et al., 2015). This implies that the initial bout of 360 shocks lays down a kind of memory for regularity that is preserved over time (a savings effect). Further, an initial bout of FT stimulation has a lasting effect independent of whether the second bout is applied at the same, or a different, locations (leg and tail; Lee et al., 2015). This suggests that a central (spinal) system can integrate inputs from distinct dermatomes.

Our prior work indicates that spinal systems can discriminate FT and VT stimulation and that this requires extended training (540 or more shocks; Baumbauer et al., 2008; Lee et al., 2015). This observation, together with evidence of savings, the lasting nature of the protective effect, and its dependance upon the NMDAR and protein synthesis (Baumbauer et al., 2009; Lee et al., 2015), implies that a form of learning is involved. We have suggested that the ability to identify a stimulus as regular may depend upon the central pattern generator (CPG) that drives the tempo of stepping. However, our evidence for this is slim, based largely on the fact that both stepping and learning about FT stimulation occur within a similar frequency range and stimulus parameters (Roy et al., 1991; de Leon et al., 1994; Cha et al., 2007). Indeed, at present, we have no evidence to discount an hourglass model of our effects. Further, the discrimination between FT and VT stimulation may not require spinal processing; if FT stimulation engages a distinct set of afferent fibers, a sensory signal could provide a *peripheral cue* that the stimuli occur at regular temporal intervals (Figure 1A). While it might be thought that integration across dermatomes discounts this possibility, what could be integrated is simply a sensory output indicative of regularity (an *index of regularity*). In contrast, a central account assumes that spinal mechanisms mediate *both* the abstraction of regularity *and* its integration across dermatomes (Figure 1B).

We begin by examining whether timing involves a peripheral cue or a central timer. We then explore whether timing is mediated by a kind of neural hourglass or an oscillator. Our findings suggest a central process is at work and that this spinal mechanism is linked to the oscillatory system that drives the rhythmicity of stepping behavior.

## Materials and Methods

### Subjects

Subjects were male Sprague-Dawley rats obtained from Harlan (Houston, TX, USA) that were approximately 100–120 days old, and between 300 and 400 g. All subjects were pair housed and maintained on a 12 h light/dark cycle, with all behavioral testing performed during the light cycle. Food and water was available *ad libitum*. All experiments were carried out in accordance with National Institutes of Health (NIH) standards for the care and use of laboratory animals (NIH publications No. 80–23), and were approved by the University Laboratory Animal Care Committee at Texas A&M University. Every effort was made to minimize suffering and limit the number of animals used.

### Spinal Cord Transection

Before surgery, the fur over the surgical site was shaved and disinfected with betadine solution (H-E-B, San Antonio, TX, USA). Subjects were anesthetized with isoflurane gas. Anesthesia was induced at 5% isoflurane and maintained at 2–3% isoflurane. Each subject’s head was rendered immobile in a stereotaxic apparatus and a small (5.0 × 4.0 × 2.5 cm) gauze pillow was placed under the subject’s chest to provide support for respiration.

Subjects received a complete transection at the second thoracic vertebrae (T2). After an anterior to posterior incision was made, the tissue just rostral to T2 was cleared using rongeurs, and the spinal cord exposed and cauterized. The remaining gap in the cord was filled with Gelfoam (Pharmacia Corp., Kalamazoo, MI, USA) and the wound was closed with Michel clips (Fisher Scientific, Waltham, MA, USA).

To transect the spinal cord at T12 or L3, a 7 cm anterior to posterior incision was made and the tissue above and lateral to T13-L2 was removed. After a laminectomy, the tissue was transected with a knife cut. The wound was then closed with Michel clips.

Following closure of the wound, the surface of each leg was shaved for electrode placement. Intraperitoneal (i.p.) injections (3 mL) of 0.9% saline solution were administered post-operatively to prevent dehydration. Following surgery, rats were placed in a temperature-controlled environment (25.5°C) and monitored until awake. All rats were checked every 6–8 h during the 18–24 h post-surgical period. During this time, hydration was maintained with supplemental injections of saline and the rats’ bladders and colons were expressed as needed.

Spinal transections at T2 were confirmed by (a) inspecting the cord at the time of surgery; (b) observing the behavior of the subjects after they recovered to ensure that they exhibited paralysis below the level of the forepaws and did not exhibit any supraspinally-mediated pain responses to leg shock; and (c) by visually examining the tissue postmortem.

### Stimulation Procedures

Stimulation was applied while subjects were restrained in Plexiglas tubes (23.5 cm long × 8.0 cm internal diameter). The front of each tube was sealed and the tubes were painted black, providing a dark enclosure in which rats could rest undisturbed. Holes were drilled into the anterior portion of the tubes to allow for ventilation. Two slots were cut 4 cm apart and 1.5 cm from the posterior end of the tube to allow both hind legs to hang freely. The dimensions of this apparatus were designed to loosely restrain the subjects, to keep them in place with minimal stress. Behaviorally, subjects exhibit no signs of struggling and appear to rest comfortably, unaware of the stimulation applied to dermatomes caudal to the spinal transection.

Tailshock was administered through electrodes constructed from a modified fuse clip. The electrode was coated with Spectra electrode gel (Harvard Appartus, Holliston, MA, USA) and secured with tape approximately 5 cm from the base of the tail. Subjects were loosely restrained in the Plexiglas tubes described above. A constant current 1.5 mA shock was delivered using a 660-V transformer. Shock onset and offset were controlled by the computer.

Legshock was administered to the tibialis anterior muscle. Prior to testing, the area over the muscle was shaved. An electrode constructed from a stainless steel wire (0.05 mm^2^ [30 AWG]) was inserted through the skin over the tibia, 1.5 cm from the tarsus. A second electrode made from a fine wire (0.01 mm^2^ [36 AWG], magnet wire single beldsol) was inserted perpendicular to the leg, through the body of the tibialis anterior muscle, 1.7 cm above the first electrode. The electrodes were connected to a constant current AC shock generator (Model SG-903; BRS/LVE, Laurel, MD, USA) and shock intensity was adjusted to a level that produced a 0.4 N flexion response, as described in Grau et al. (1998).

Custom software running on a Macintosh computer was used to control the presentation of shock. FT shocks were 80 ms in duration and occurred at a regular interval [a fixed inter-stimulus interval (ISI)] that was set to 2 s (0.5 Hz). VT shocks of the same duration were presented using a variable ISI that ranged from 0.2–3.8 s (rectangular distribution) with a mean of 2 s.

### Instrumental Testing

Testing was conducted while subjects were loosely restrained in the Plexiglas tubes (Figure 2B), with their hind legs hanging freely over a salt bath (NaCl). Leg shock was delivered to the tibials anterior muscle as described above. A contact electrode was constructed from a 7 cm long, 0.46 diameter, stainless steel rod. The contact electrode was taped to the plantar surface of the rat’s foot (Orthaletic, 1.3 cm [width]; Johnson and Johnson, New Brunswick, NJ, USA) with the end positioned directly in front of the plantar protuberance. Heatshrink tubing electrically insulated the rod from the paw. A fine wire (0.01 mm^2^ [36 AWG], magnet wire single beldsol) was attached to the end of the rod at a point under the insulation. This wire extended from the rear of the foot and was connected to a digital input board that was monitored by the Macintosh computer. To minimize lateral leg movements, a piece of porous tape (Orthaletic, 1.3 cm [width]) was wrapped around the leg above the tarsus and attached under the front panel of the restraining tube. A rectangular plastic dish (11.5 cm [w] × 19 cm [l] × 5 cm [d]) was positioned 7.5 cm below the restraining tube and filled with a NaCl solution. A drop of soap was added to reduce surface tension. A ground wire was connected to a 1 mm wide stainless steel rod, which was placed in the solution. Three short (0.15 s) shock pulses were applied and the level of the salt solution was adjusted so that the tip of the contact electrode was submerged 4 mm below the surface. Subjects then received 30 min of response contingent shock (instrumental testing). When the contact electrode touched the underlying salt solution, shock was delivered to the tibialis anterior muscle causing the ankle to flex, lifting the contact electrode out of the salt solution and terminating the shock.

Leg position was monitored using a Macintosh computer at a sampling rate of 30 Hz. Performance was measured over time in 30, 1 min time bins. The computer monitoring leg position and recorded an increase in response number whenever the contact electrode was raised above the salt solution. Response duration within each 1 min bin was calculated using the following equation: Response Duration = (60 s − time in solution)/(Response Number + 1). Subjects capable of instrumental learning exhibit a progressive increase in response duration that minimizes net shock exposure (Grau et al., 1998).

To evaluate whether our experimental treatment affected baseline behavioral reactivity, we analyzed both the shock intensity required to elicit a flexion force of 0.4 N and the duration of the first shock-elicited flexion response. Independent ANOVAs showed that there were no group differences on either measure across all experiments (*F*’s < 1.63), *p* > 0.05.

### Statistics

All data were analyzed using repeated measures analysis of variance (ANOVA). When necessary, *post hoc* comparisons of the group means were performed using Duncan’s New Multiple Range test. In all cases, a criterion of *p* < 0.05 was used to judge statistical significance.

### General Experimental Design

Experimental treatments commenced a day after subjects received a complete spinal transection. Experiment 1 examined the impact of regular stimulation (720 shocks) distributed across two dermatomes (leg and tail; Figure 2A) in an alternating, concurrent, or random manner. Experiment 2 applied stimuli to two dermatomes at the same, or slightly different, frequencies to produce either a coherent, or incoherent pattern of stimulation. Experiment 3 evaluated the effect of randomly omitting half the stimuli from a train of 720 FT or VT tailshocks. In all three experiments, shock treatment was followed by 30 min of testing in the instrumental learning paradigm. Experiment 4 examined whether randomly omitting shocks affects the long-term protective effect of FT stimulation. A day after treatment, subjects received 180 VT shocks and then underwent 30 min of instrumental testing. Experiment 5 evaluated whether shocks must remain in phase after stimulus omission. Finally, Experiment 6 assessed whether surgically disconnecting the region that drives the tempo of stepping (L1-L2) from the area that mediates learning (L4-S2) would affect the abstraction of regularity. In both Experiment 5 and 6, instrumental learning was tested immediately after shock treatment.

## Results

### Experiment 1: Regularity can be Abstracted When the Site of Stimulation is Varied

Regularity could be detected by means of a peripheral cue or a central process. The latter spinal mechanism abstracts regularity from the afferent input. If this central process has access to multiple inputs, regularity could be abstracted when the site of stimulation is varied. In contrast, the peripheral account posits that regular and irregular stimuli engage different sets of afferent nerves (Figure 1B). Because the learning deficit depends upon C-fiber activity, and because 180–360 FT shocks impair subsequent learning, we assume that both FT and VT stimulation induce C-fiber activity (for additional discussion of the relation of these effects to nociceptive sensitization and wind-up, see Ferguson et al., 2006, 2012; Baumbauer et al., 2008; Joynes et al., 2004; Hook et al., 2008). Given this, a sensory cue for regularity could emerge if FT stimulation uniquely activates an alternative fiber type (e.g., A-fiber activity), providing a cue indicative of regularity. From this perspective, spinal systems simply monitor the relative strength of this index of regularity, summating its value across time and location (dermatome); once a threshold value is reached, which requires 540 or more shocks (Lee et al., 2015), the restorative effect of FT stimulation would be engaged.

**Figure 3:**
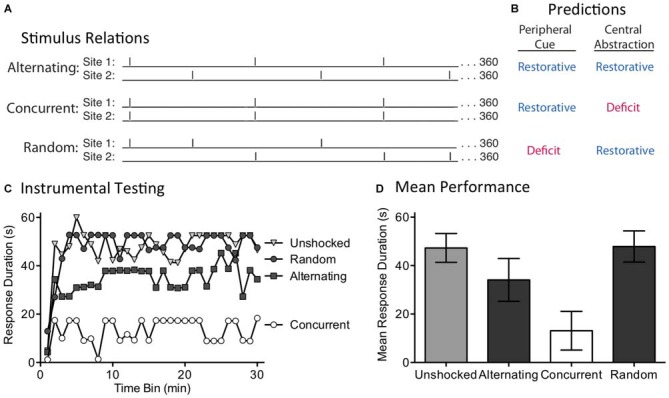
**The induction of the restorative effect requires a regular pattern of stimulation across dermatomes. (A)** Spinally transected rats received 360 shocks to two dermatomes [leg and tail (site 1 and 2)] at 0.25 Hz. Shocks were presented in an alternating or concurrent fashion. In the random condition, stimulus location was randomly varied over time. **(B)** The peripheral cue and central abstraction models differ in whether these treatments should reinstate the capacity for learning (blue) vs. induce a learning impairment (red). **(C)** Next, subjects underwent 30 min of testing with response-contingent shock applied to the untreated hindleg. Only the concurrent condition produced a learning impairment, as predicted by the central account. **(D)** Mean test performance [± the standard error of the mean (SEM)].

The present experiment explores these alternatives by concurrently applying 360 FT shocks to two sites (leg and tail) and varying their temporal relation (Figure 3A). For two groups, shocks were given at 0.25 Hz (4 s apart) at regular intervals. One group received the shocks concurrently. The other received the shock in an alternating manner, generating a stimulus train that had an overall frequency of 0.5 Hz. The last group also received shock to two dermatomes at 0.5 Hz, but the locus of stimulation was randomly varied over time. A peripheral account assumes that regular stimulation to two dermatomes will engage a sensory cue indicative of regularity, which would then be summed across dermatomes. Because this system is insensitive to the phase relation across dermatomes, it should not matter whether the shocks are given in an alternating or concurrent manner—both should produce a restorative effect (Figure 3B). For a peripheral cue to be engaged, the shocks applied to each dermatome must be regular in nature. For this reason, randomly varying where shock is applied should weaken the sensory cue and undermine the development of the restorative effect. If a central process is at work, and can abstract regularity across dermatomes, both the alternating and random conditions should generate a restorative effect. Because the concurrent condition only generates (centrally) 360 pulses of stimulation (too few to induce a restorative effect), it should produce a learning deficit.

We evaluated these predictions in rats that had received a spinal transection at T2. A day after surgery, subjects (*N* = 32) were placed in restraining tubes and set up to receive shock to the tail and one hind limb (site 1 and 2; counter-balanced across subjects). Over the next 24 min, separate groups received shock to the tail and leg or nothing (Unshocked). Two of the shocked groups received 360 FT shocks to each dermatome at 0.25 Hz (4 s ISI). For one group, the shocks occurred at the same time (Concurrent) while for the other the shocks alternated (Alternating). A third shocked group (Random) received a shock stream wherein stimuli occurred at 0.5 Hz for 24 min, but the locus of stimulation was randomly varied from trial to trial. At the end of shock treatment, subjects were set up for instrumental testing on the untreated hindlimb. All subjects then received 30 min of testing with response-contingent shock.

As expected, spinally transected rats that were unshocked prior to testing exhibited a progressive increase in flexion duration indicative of instrumental learning (Figure 3C). Concurrent exposure to 360 shocks induced a learning impairment. When the shocks were given in a manner that yielded an alternating pattern across dermatomes, the capacity for learning was restored. This remained true when the site of stimulation was randomly alternated over time. An ANOVA confirmed that shock treatment had a significant effect [*F*_(3,28)_ = 4.92], *p* < 0.01. The main effect of time bin [*F*_(29,812)_ = 7.81], and the Time Bin × Shock Treatment interaction [*F*_(87,812)_ = 1.57], were also significant, *p* < 0.005. *Post hoc* comparisons of the group means (Figure 3D) showed that the concurrent group differed from the other three, *p* < 0.05. No other comparison was significant, *p* > 0.05.

If the abstraction of regularity involves a peripheral cue, and the output of this process is simply summed across dermatomes, both concurrent and alternating stimulation should have had an analogous effect. The fact two bouts of shock only had a restorative effect when stimuli were presented in an alternating or random manner suggests that a central process may be at work.

### Experiment 2: The Abstraction of Regularity Requires a Coherent Pattern Across Dermatomes

**Figure 4:**
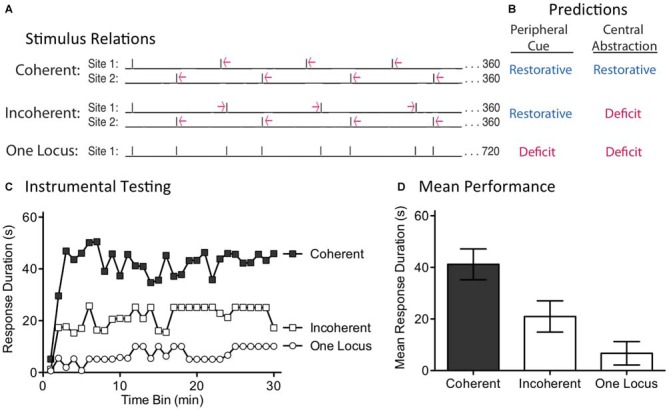
**Disrupting the phase relation across dermatomes eliminates the restorative effect. (A)** Two groups received shock to one hind leg and the tail (site 1 and 2). Shock frequency was set to 0.256 or 0.243 Hz. When the frequency applied to both sites was the same, a coherent alternating relation was maintained. When the frequency of stimulation across dermatomes differed, the relation across dermatomes varied (Incoherent). A third group received the same pattern of stimulation as the Incoherent group, but to a single site. Subjects then received 30 min of instrumental testing. **(B)** If a peripheral cue underlies timing, the relation across dermatomes should not matter. As a result, both the Coherent and Incoherent condition should have a restorative effect (blue). If a central process abstracts regularity across stimulation sites, the Incoherent relation should yield a learning deficit (red). **(C)** Subjects then underwent 30 min of instrumental testing with response-contingent stimulation applied to the untreated leg. Only the Coherent condition yielded a restorative effect, as predicted by the central account. **(D)** Mean performance collapsed across time bins (±SEM).

Our first experiment discounted a simple version of timing based on a peripheral cue. One could argue, though, that this provided an unfair test because concurrent stimulation might disrupt the integration of regularity across dermatomes. Further, the semi-regular pattern of shock applied to each dermatome in the Random condition may have been sufficient to engage the putative sensory cue. For these reasons, a stronger test is needed. We address this issue by shifting the temporal relationship across sites over time (Figure 4A). This was accomplished by presenting regular stimulation to each dermatome, but at slightly different (±100 ms) ISIs. This generated trains of shock that differ by a small amount in frequency (0.256 vs. 0.243 Hz). In all cases, 360 shocks were applied to each dermatome and the second shock stream began at the mid-point of the first ISI. Under these conditions, if the stimuli applied to both sites have the same frequency, the pattern across dermatomes will continue to alternate in a regular (Coherent) manner. If, however, the stimuli applied to each dermatome have a different frequency, the temporal relation will shift (Incoherent) over time. A central process should only be able to abstract regularity when the stimuli maintain a coherent relation across time. In contrast, if regularity is abstracted by a peripheral process, the relation across dermatomes should not matter. Under these conditions, both the Coherent and Incoherent pattern should produce the same effect; both should have a restorative effect (Figure 4B). As a positive control, a third group received the incoherent pattern of stimulation, but to a single dermatome. Our prediction was that this irregular shock pattern would generate a learning impairment.

We examined these predictions in spinally transected rats (*N* = 36). A day after surgery, subjects were placed in restraining tubes and set-up to receive leg and tail shock (site 1 and 2). One third then received regular shock to each dermatome at the same frequency, either 0.256 Hz (ISI = 4.1 s) or 0.243 Hz (ISI = 3.9 s). The second shock train started at the temporal mid-point of the first ISI, generating an alternating (Coherent) pattern of shock across time. Another third received shock to each dermatome, but at different frequencies (0.256 and 0.243 Hz). Under these conditions, the phase relation between each shock train will slowly rotate across time, producing a pattern of shocks wherein the temporal gaps contract and expand (Incoherent). For both groups, which dermatome was stimulated first, and the ISI employed for stimuli applied to each dermatome, was counter-balanced across subjects. The last third received the same incoherent pattern of shocks, but applied to a single dermatome (One Locus; leg or tail, counter-balanced across subjects). At the end of the stimulation period (approximately 24 min), subjects were set-up for instrumental testing on the untreated leg. They were then tested for 30 min with response-contingent shock.

When subjects received regular shock to each dermatome, but at different frequencies, the resultant incoherent pattern of shock produced a learning impairment (Figure 4C). A similar effect was observed when this same incoherent pattern was applied to a single dermatome (One Locus). In contrast, when the shocks were applied at the same frequency across dermatomes, the resultant alternating shock pattern restored the capacity to learn. An ANOVA showed that the main effects of shock treatment [*F*_(2,33)_ = 8.84] and time bin [*F*_(29,957)_ = 3.49] were significant, *p* < 0.001. *Post hoc* comparisons of the group means (Figure 4D) confirmed that the Coherent group differed from the other two, *p* < 0.05. No other comparison was significant, *p* > 0.05.

Using identical shock trains applied to two locations, we showed that training only had a restorative effect when the relation across dermatomes was regular (Coherent). When the phase relation between the shocks was manipulated across dermatomes, the resultant incoherent pattern of stimulation induced a learning impairment. These results discount a peripheral account of our results and show that regularity is abstracted by a central process. At the same time, the results also reveal some limits to this process. For example, if the central system was capable of performing a kind of Fourier analysis (e.g., through a bank of oscillators, each tuned to a particular frequency), the embedded regular signals (within both the Incoherent and One Locus conditions) could have been abstracted. Likewise, rotating the phase relation across dermatomes generated a predictable, but complex, pattern of stimulation. Our results suggest that, under the current training conditions, spinal systems are incapable of these more sophisticated forms of timing.

### Experiment 3: Filling in What is Missing: Abstracting Regularity When Stimuli are Omitted

**Figure 5:**
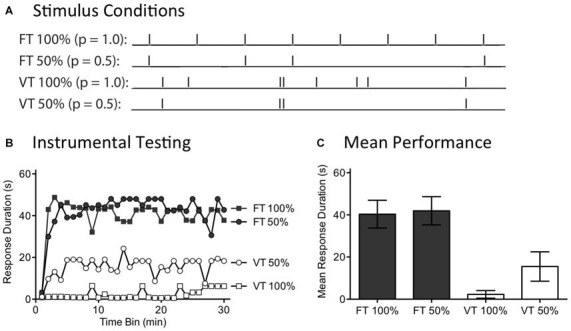
**Randomly omitting half the shocks does not affect the restorative effect of fixed time (FT) stimulation. (A)** Spinally transected rats received tail shock for 24 min on a variable time (VT) or FT schedule. For two groups, half the shocks (50%) were randomly omitted. **(B)** Subjects were then tested for 30 min with response contingent leg shock. FT stimulation had a restorative effect when half the stimuli were randomly omitted. **(C)** Mean test performance (±SEM).

Our results indicate that a spinal mechanism underlies the abstraction of regularity. Researchers within the timing literature have related this type of effect to two potential mechanisms. One assumes a biological process (a neural timer) that decays over a set period of time, providing a kind of *hourglass* (Boulos and Terman, 1980). Any biological process that decays in a regular manner (e.g., phosphorylation state, protein binding, chemical diffusion) could provide the foundation for a neurobiological timer. The alternative view links the abstraction of regularity to a cyclic device (an endogenous *oscillator*) that generates an output at the same frequency as the external stimulus. It is often assumed that internal oscillators have a sort of momentum that can drive the process after environmental stimulation has ended (Grillner and Zangger, 1979; Grillner et al., 1991; Kiehn, 2006; Hultborn and Nielsen, 2007; Rossignol and Frigon, 2011; Frigon, 2012). For example, models of circadian timing have provided evidence for an internal oscillator that can continue to drive waking/sleeping behavior when subjects are maintained in a dark environment (Mauk and Buonomano, 2004; Herzog, 2007; Ivry and Schlerf, 2008). Likewise, we posit that exposure to regular stimulation could involve a spinal oscillator, possibly related to the CPG thought to underlie the generation of rhythmic stepping. Like a circadian oscillator, the CPG linked to locomotion has a kind of momentum that helps to maintain the phase of stepping over time (Grillner, 1973; Grillner and Zangger, 1979; Grillner et al., 1991; Kiehn, 2006; Rossignol and Frigon, 2011). If this type of process contributes to the abstraction of regularity, it may continue to work even when some stimuli are omitted. The present experiment explores this possibility by evaluating the effect of randomly omitting half the shocks from a train of 720 (Figure 5A). Normally, 360 fixed spaced shocks induce a learning impairment (Lee et al., 2015). The novel prediction is that omitting shocks from a series of fixed spaced stimulation will have a negligible effect and for this reason, 360 physical shocks should have the same restorative effect as 720 shocks. We also tested whether randomly omitting half the shocks from a train of 720 variably spaced stimuli impacts the development of the learning impairment.

A day after spinal transection, rats (*N* = 40) were randomly assigned to four conditions. All subjects were placed in restraining tubes and had tail electrodes attached. One group received 720 tail shocks (80 ms) spaced 2 s apart (FT 100%). Another received stimulation over the same period of time, but half the shocks were randomly omitted (FT 50%). A third group (VT 100%) was given shock on variable schedule (0.2–3.8 s; mean ISI = 2 s) while a fourth group received variable shock with half the stimuli randomly omitted (VT 50%). For those conditions with omitted stimuli, the software randomly determined whether to present a shock on-line on a trial-by-trial basis. Immediately after the last shock, instrumental learning was tested for 30 min.

As expected, exposure to variable shock (VT 100%) induced a learning impairment (Figure 5B). Omitting half the shocks from this schedule (VT 50%) did not significantly diminish the learning impairment. No learning impairment was observed in rats that received 720 shocks on a fixed ISI (FT 100%). Most importantly, this remained true when half the shocks were randomly omitted (FT 50%). An ANOVA confirmed that the outcome observed depended upon whether rats received fixed or variable shock [*F*_(1,36)_ = 26.41], *p* < 0.001. Additionally, the Time Bin × Shock schedule interaction was significant, [*F*_(87,1044)_ = 1.68], *p* < 0.001. Stimulus omission did not have a significant effect on test performance [*F*(_29,1044_) = 1.27], *p* > 0.05. *Post hoc* comparisons of the group means (Figure 5C) showed that the two FT treated groups differed from the two groups given VT shock, *p* < 0.05. No other comparison was significant, *p* > 0.05.

In our companion article (Lee et al., 2015), we show that 360 shocks given on a FT schedule induce a lasting learning impairment and that this is true independent of whether the stimuli occur at a frequency of 1, 0.5, or 0.25 Hz. Likewise, we reported above that concurrent shocks given at 0.25 Hz produce a learning impairment. In all these cases, a deficit was observed when a continuous stream of shocks was administered. The present experiment evaluated another method of applying 360 shocks, generated by randomly omitting half the stimuli from a train of 720 FT stimuli. What was surprising is that omitting shocks did not eliminate the restorative effect of stimulation, even though only 360 physical shocks were applied. The results suggest that a central system effectively “fills-in” the missing stimuli. The capacity to do this naturally fits with the idea that the abstraction of regularity is coupled to an internal oscillator that becomes entrained to the stimulus and that continues to cycle when stimuli are omitted.

### Experiment 4: Stimulus Omission does not Undermine the Long-Term Effect of Regular Stimulation

**Figure 6:**
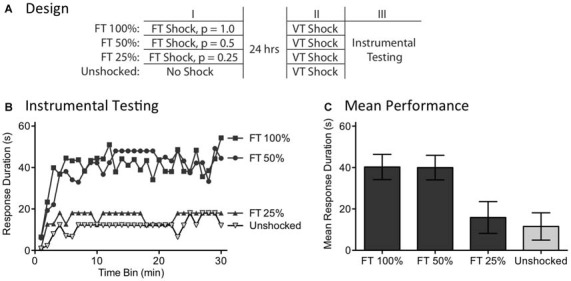
**Randomly omitting half the shocks does not undermine the long-term protective effect of FT stimulation. (A)** One group received 720 tail shocks on a FT schedule (FT 100%). Other groups had 1/2 (FT 50%) or 3/4 (FT 25%) of the shocks randomly omitted. A final group (Unshocked) received nothing during the first session. A day later, all subjects received 180 tail shocks on a VT schedule, followed by 30 min of instrumental testing. **(B)** During instrumental testing, subjects that had previously received nothing (No Shock) prior to VT stimulation exhibited a learning impairment. Prior exposure to FT shock blocked the learning deficit and this protective effect survived omission of half, but not 3/4, of the stimuli. **(C)** Mean performance collapsed across the 30 min of testing (±SEM).

Recognizing the surprising nature of the above results, we sought further evidence that the effects of fixed spaced stimulation can develop when stimuli are omitted. We addressed this issue by examining the long-term effect of FT shock treatment. We have previously shown that exposure to 720 fixed spaced shocks (FT 100%) induces a lasting modification within the spinal cord that blocks the induction of the learning impairment when rats are exposed to variable shock 24 h later (Baumbauer et al., 2009). Here, we asked whether fixed spaced shock has a lasting protective effect when half the shocks are randomly omitted (FT 50%; Figure 6A). We also evaluated the limits of this process by testing the impact of omitting more (3/4) shocks (FT 25%).

A day after rats received a spinal transection (*N* = 40), they were randomly assigned to four experimental conditions. All subjects were placed in restraining tubes and had tail electrodes attached. One group (FT 100%) received 720 shocks (80 ms) spaced 2 s apart. Another received stimulation over the same period of time, but half the shocks were randomly omitted (FT 50%). A third group had 3/4 of the shocks randomly omitted (FT 25%) and the final group received no shock (Unshocked). The rats were then returned to their homecages. The next day, they were placed in the restraining tubes and had the tail electrodes attached. All rats received 180 shocks on a variable (0.2–3.8 s) schedule (average ISI = 2 s). Subjects then had leg electrodes attached to the untreated leg and were set-up for instrumental testing. Finally, response-contingent shock was applied for 30 min.

As usual, rats that had previously received no shock (Unshocked) exhibited a learning impairment when given VT shock (Figure 6B). Prior treatment with 720 fixed spaced shock (FT 100%) had a protective effect that blocked the induction of the learning impairment. Omitting half the shocks (FT 50%) had no effect, but omitting 3/4 (FT 25%) eliminated the protective effect. An ANOVA showed that the initial shock treatment had a significant effect [*F*_(3,36)_ = 4.88], *p* < 0.01. The main effect of time bin was also significant [*F*_(29,1044)_ = 6.16], *p* < 0.0001. *Post hoc* comparisons of the group means (Figure 6C) revealed that subjects given 100 and 50% FT shock differed from those given FT 25% or nothing (Unshocked), *p* < 0.05. No other differences were significant, *p* > 0.05.

As previously reported, exposure to fixed spaced shock blocked the induction of the learning impairment when subjects were later challenged with variable shock (Baumbauer et al., 2009; Lee et al., 2015). Here, we showed that this effect is induced when half the shocks from a train of 720 are randomly omitted. These findings provide further evidence that spinal systems can fill-in missing stimuli. The results also revealed a limit to this process, showing that no protective effect is observed when more shocks (3/4) are omitted. It is, of course, possible that a FT effect would emerge with this schedule if subjects were given additional training.

### Experiment 5: Missing Signals are Only Filled in if the Stimuli Remain in Phase

**Figure 7:**
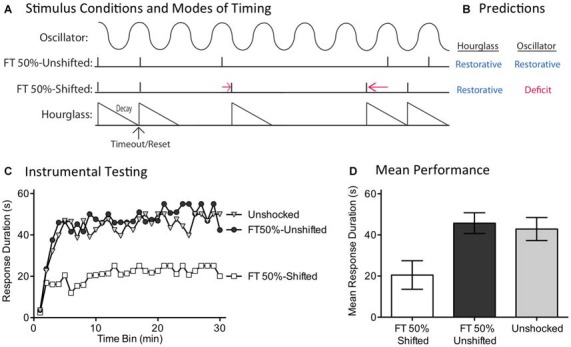
**Disrupting the phase relation after an omitted stimulus eliminates the restorative effect of FT stimulation. (A)** Spinally transected rats received nothing (Unshocked) or FT stimulation (tail shock) with half the shocks randomly omitted. For half the shocked rats (FT 50%-Unshifted), all physical shocks remained in phase. For subjects in the FT 50%-Shifted condition, the onset of a shock after stimulus omission was randomly varied from trial to trial, which shifted the phase relation. **(B)** Only the account based on an internal oscillator predicts that shifting the phase relation will alter the effect of shock treatment, eliminating the restorative effect. **(C)** Subjects were then tested with response-contingent leg-shock. A restorative effect was only observed when the shocks remained in phase (FT 50%-Unshifted). **(D)** Mean performance collapsed across the 30 min of testing (±SEM).

While the capacity to fill-in missing stimuli fits well with a model built upon an endogenous oscillator, we recognized that some versions of an hourglass model could accommodate this finding. For example, inserting a temporal gap between stimuli provides a form of spaced practice that could potentiate the amount that is learned when the next (unexpected) physical stimulus is presented. If this is true, fewer iterations would be needed to learn about regularity. Under these conditions, half the number of shocks (FT 50%) might restore the capacity to learn. For this reason, discounting an hourglass model requires a stronger test. We address this issue by evaluating a core feature of an oscillatory system—the idea that regularity is linked to the synchrony between the physical stimuli and an internal oscillator. For this system to work in the face of missing stimuli, the physical stimuli must remain in phase with the hypothesized oscillator (Figure 7A). This is not required for a system built upon an hourglass-like timer. Here it is assumed that the physical stimulus triggers an endogenous process that decays over the ISI, providing a biological marker of elapsed time that is stamped in by the presentation of the next physical stimulus. But if a physical stimulus is omitted, the timer is not restarted. Minus the timer, there is no cue to indicate when the next physical stimulus should occur. For an hourglass model, stimuli across bouts need not remain in phase; all that matters is that the stimuli within a physical bout of shocks occur at the same ISI. For these reasons, only the hourglass model predicts that both the shifted and unshifted conditions will have a restorative effect (Figure 7B).

We evaluated these alternative predictions in spinally transected rats (*N* = 36), A day after surgery, subjects were placed in the restraining tubes and tail electrodes attached. One group (FT 50%-Unshifted) received a train of 720 fixed spaced tailshocks (ISI = 2 s) with half the stimuli randomly omitted. A second group (FT 50%-Shifted) was trained in a similar manner, except the time at which shocks resumed after an omission was randomly varied between 0.2 and 3.8 s (mean = 2 s). All subsequent shocks, until the next omission, were presented on an ISI of 2 s. For each subject in this group, which stimuli were omitted, and the value of the delay, were randomly determined (given the above constraints) on-line. A third group (Unshocked) received no shock. Next, subjects were set-up to receive response-contingent shock to the left or right hind leg (counter-balanced across subjects) and underwent 30 min of instrumental testing.

As expected, previously untreated (Unshocked) rats exhibited a progressive increase in flexion duration over the 30 min of testing (Figure 7C). Rats that had received a train of fixed spaced stimulation, with half the shocks omitted and no phase shift (FT 50%-Unshifted), also learned. When phase was randomly shifted after an omission (FT 50%-Shifted), shock treatment induced a learning impairment. An ANOVA confirmed that shock treatment had a significant effect [*F*_(2,33)_ = 5.02], *p* < 0.05. The main effect of time bin was also significant [*F*_(29,957)_ = 10.66], *p* < 0.0001. *Post hoc* comparisons of the group means (Figure 7D) showed that the FT 50%-Shifted group differed from the other two, *p* < 0.05. The Unshocked and FT 50%-Unshifted groups did not differ, *p* > 0.05.

If an hourglass-like device underlies the identification of a stimulus train as regular, shifting the phase of the stimuli after an omitted shock should have little effect. Contrary to this prediction, we found that phase-shifted rats exhibited a learning impairment, implying that this treatment disrupted the derivation of regularity. This pattern of results again suggests that timing depends upon a spinal oscillator.

An hourglass model could potentially be augmented to handle the present results. For example, it might be argued that training involves a biological process that indicates the end of the timed period and re-engages the timer. However, in positing that the hourglass acquires the capacity to re-start itself, we have effectively constructed a kind of cyclic device (oscillator). Conceptually, the primary difference would lie in the role of training: does training serve to engage a pre-existing oscillator or create a new neuronal system that cycles at the appropriate tempo? Further work is needed to distinguish these alternatives.

### Experiment 6: Surgically Disrupting the Capacity to Abstract Regularity

**Figure 8:**
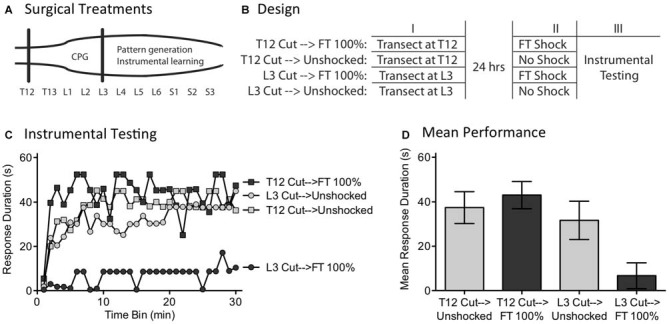
**A L3 transection eliminates the restorative effect of FT stimulation. (A)** Subjects underwent a spinal transection either rostral (at T12) or caudal (at L3). **(B)** The next day, subjects received FT stimulation or nothing, followed by 30 min of instrumental testing. **(C)** When tested with response contingent shock, FT stimulation induced a learning impairment in subjects that had undergone a L3 transection. **(D)** Mean performance collapsed across the 30 min of testing (±SEM).

We have previously shown that instrumental learning is mediated by neurons within the lower lumbosacral (L4-S2) spinal cord (Liu et al., 2005). Interestingly, a more rostral system (L1-L2) appears to drive the pace (tempo) of stepping (Figure 8A; Cazalets et al., 1995; Magnuson et al., 1999, 2005). Supporting this, neurons within the L1-L2 region have been shown to exhibit cyclic activity (Kiehn and Kjaerulff, 1998; Kiehn, 2006). Further, localized lesions of the L1-L2 central gray disrupt rhythmic stepping behavior (Magnuson et al., 1999). These observations imply the L1-L2 region plays an essential role in driving the tempo of stepping, either because the key oscillator lies within this area or because neurons within it drive a caudal oscillatory network. Here, we explore whether the L1-L2 lumbar tissue is also essential to the abstraction of regularity in response to fixed spaced stimulation. If it is, surgically disconnecting this region from the lower (L4-S2) tissue should disrupt spinal timing. We tested this hypothesis by transecting the spinal cord rostral (at T12) or caudal (L3) to the L1-L2 region prior to FT stimulation or nothing (Figure 8B). Because spinal learning is mediated by neurons caudal to L3, neither transection should disrupt instrumental learning. The novel prediction is that cutting the spinal cord at L3 will eliminate the ability to abstract regularity. Under these conditions, an extended exposure to fixed spaced shock should not have a restorative effect and produce a learning impairment.

Subjects (*N* = 32) underwent a spinal transection rostral to T12 or at L3. The next day, they were placed in restraining tubes and had tail electrodes attached. Half the subjects in each condition then received 720 fixed spaced shocks (FT 100%) with an ISI of 2 s (0.5 Hz). The remaining subjects received no shock (Unshocked). Subjects were then set-up for instrumental testing on the left or right hind leg (counter-balanced across subjects) and received 30 min of response-contingent shock.

As expected, unshocked rats that received either a transection at T12 (T12 Cut→Unshocked) or L3 (L3 Cut→Unshocked) exhibited a progressive increase in flexion duration over the course of the 30 min of testing (Figure 8C). Rats that received a T12 transection and fixed spaced stimulation (T12 Cut→FT 100%) were also able to learn. However, rats given fixed spaced shock after a L3 transection (L3 Cut→FT 100%) exhibited a learning impairment. An ANOVA confirmed that the main effect of surgery [*F*_(1,28)_ = 8.24] and Surgery × Shock treatment interaction [*F*_(1,28)_ = 4.38] were statistically significant, *p* < 0.05. So too was the main effect of time bin [*F*_(29,812)_ = 4.73], *p* < 0.0001. *Post hoc* comparisons of the group means (Figure 8D) showed that subjects that received a cut at L3 prior to FT shock differed from all other groups, *p* < 0.05. No other group difference was significant, *p* > 0.05.

As previously reported, we found that a transection at L3 does not disrupt spinally-mediated instrumental learning. It did, however, modify how fixed spaced stimulation affects spinal function; instead of restoring the capacity to learn, presenting 720 fixed space shocks induced a learning impairment. It appears that neurons rostral to L3 are needed to drive regular stepping (Cazalets et al., 1995; Magnuson et al., 1999) *and* abstract regularity in response to cutaneous stimulation. The results also suggest that the learning impairment is mediated by neurons caudal to L3, presumably within the same region (L4-S2) implicated in spinal learning.

## Discussion

### Temporal Regularity is Abstracted by a Central Process

Prior work has shown that fixed and variably spaced intermittent stimulation has opposite effects on spinal plasticity; 720–900 shocks given in a variable manner induce a lasting learning impairment whereas this same number of shocks given on a fixed ISI induces a protective/restorative effect that counters the adverse effect of variable stimulation (Baumbauer et al., 2009). What was not known is how lower level systems are able to discriminate fixed from variable stimulation. We recognized that regularity could be abstracted by means of a peripheral cue or a central (spinal) process (Figure 1B). We evaluated these alternatives by varying the phase relationship between stimuli applied to two dermatomes. From prior work we knew that exposure to two bouts of 360 FT shocks (at 0.5 Hz) restores the capacity to learn and that this is observed when the stimuli are applied to different dermatomes (tail and leg; Lee et al., 2015). Here, we showed that a similar result is obtained when 360 shocks are applied to each dermatome at a lower frequency (0.25 Hz) in an alternating manner (Experiment 1).

Both the peripheral and central model predicted that alternating stimuli across dermatomes would have a restorative effect indicative of timing. A peripheral account handles this observation by assuming each input engages a cue related to regularity that is centrally summed across dermatomes. A purely central account assumes that both the abstraction of regularity and the integration of this effect over time/location are spinally mediated. To evaluate these alternatives, we manipulated the phase relation between the stimuli. If a peripheral signal underlies the discrimination, the phase relation across dermatomes should not matter. In contrast, a central account requires that the stimuli occur in a coherent alternating manner across dermatomes. Our results supported the central account. When stimuli were applied across dermatomes in a concurrent fashion, or when the phase relation was rotated, stimulation induced a learning impairment. Inducing a restorative effect required 720 independent pulses of stimulation and these had to occur in a manner that yielded a regular relation across dermatomes (Experiment 2). Interestingly, this remained true when the site of stimulation was randomly varied over time. Our results implicate a spinal process and suggest that this system can dynamically abstract regularity across time and location.

### The Detection of Regularity is Coupled to an Oscillator

Within the spinal cord, temporal regularity could be detected by means of either a timer (an hourglass) or an oscillator. A distinguishing feature of many oscillatory systems is that they have a kind of momentum that drives activity after the initiating stimulus has ended, yielding a pendulum-like effect. For this reason, an oscillatory system is relatively insensitive to missing stimuli. We examined this implication by randomly omitting half the shocks from a train of 720, generating a shock schedule that contained just 360 shocks. Across multiple experiments and stimulus frequencies (0.25–1.0 Hz), an uninterrupted string of 360 FT stimuli induce a lasting learning impairment (Lee et al., 2015). But when we randomly omitted half the shocks from a train of 720, it had no impact on the restorative effect of FT stimulation (Experiment 3). Nor did it impact the long-term protective effect that counters the development of a learning impairment when subjects are challenged with VT stimulation 24 h later (Experiment 4). Further degrading the temporal relation, by omitting 75% of the stimuli, did eliminate the long-term protective effect.

An hourglass model could handle the effect of stimulus omission if it assumed that the shocks that follow a missing stimulus engender greater processing. What differentiates the hourglass model is that, once it has timed out, there is no information regarding the time at which the next physical stimulus will occur. In contrast, in an oscillatory system, subsequent physical stimuli must remain in phase; if the phase relation is degraded, the system would fail to oscillate in a coherent manner and the capacity to time should deteriorate. We examined these alternatives by manipulating the phase relation of the physical stimuli after stimulus omission. The results showed that the restorative effect only survived stimulus omission when the physical stimuli remained in phase, as predicted by a model based on an internal oscillator (Experiment 5).

### Similiarities to the Processes Involved in Generating Rhythmic Stepping

Prior work has shown that the tempo of stepping depends upon neurons within the rostral lumbar (L1-L2) spinal cord (Cazalets et al., 1995; Magnuson et al., 1999, 2005; Liu et al., 2005). To explore whether this region also plays a role in the abstraction of regularity in response to fixed spaced stimulation, we applied FT or VT stimulation to subjects that had undergone a L3 transection (Experiment 6). Minus access to the L1-L2 tissue, both FT and VT stimulation induced a learning impairment. Our results are consistent with prior work demonstrating that neurons caudal to L3 support instrumental learning (Liu et al., 2005). The present findings show that nociceptive input to this region can induce a learning impairment and that neurons rostral to L3 are needed to abstract regularity.

Others have shown that spinally injured animals given behavioral training exhibit an enhancement in adaptive plasticity that promotes locomotor function (Barbeau and Rossignol, 1987; Belanger et al., 1996; Rossignol et al., 1996; de Leon et al., 1997, 1998; Van de Crommert et al., 1998; Edgerton et al., 2004; Barriere et al., 2008; Guertin, 2009; Rossignol et al., 2009; van den Brand et al., 2012). Further, the beneficial effect of physical exercise has been linked to increased expression of BDNF (Gómez-Pinilla et al., 2002, 2007). Like FT stimulation, locomotor training can attenuate behavioral signs of pain (Hutchinson et al., 2004). These observations, coupled with the present data, suggest that FT stimulation and locomotor training may benefit spinal function for a common reason—both may engage a spinal oscillator.

### Deriving How the Oscillator Operates

We know from prior work that two bouts of 360 FT shocks, separated by 24 h, produce a restorative effect. This implies that a feature of the stimulus situation was encoded during the initial bout of stimulation and preserved over time (a kind of memory). The presence of this memory is evident from the fact that prior training transforms how the second block of shocks affects spinal function (to yield a restorative effect rather than a learning impairment). We reasoned that the underlying memory might encode either the specific interval between stimuli (a form of timing we referred to as *hard timing*) or just that there was a period of regular stimulation (*soft timing*). We explored these alternatives by varying the frequency of stimulation across days (Lee et al., 2015). Two bouts of regular stimulation had a restorative effect independent of whether the frequency across days was the same or different. This suggests that a kind of soft timing is involved, wherein a marker of regularity is abstracted and stored. When integrated across days, this index of regularity triggers the processes that promote adaptive plasticity.

## Conclusions

We assume that neural clocks come in many forms, from simple hourglass-like systems that modulate neural reactivity within a specific [stimulus-response (S-R)] pathway to sophisticated, cognitive-like, processes that can be dynamically applied across a variety of situations to encode temporal relations and predict future events (Mauk and Buonomano, 2004; Herzog, 2007; Ivry and Schlerf, 2008). The latter implies a kind of memory that preserves a key environmental property. We have been able to show that the discrimination of fixed and variable stimulation impacts more than a specific S-R pathway; that regularity can be abstracted when stimuli are applied to dermatomes that are innervated by different regions of the spinal cord. In this sense, the abstraction of regularity exhibits a cognitive-like property. We have also shown that FT stimulation has a lasting effect (that counters the induction of the learning impairment by VT stimulation) and that the induction of this effect depends upon both NMDAR-mediated plasticity and protein synthesis (Baumbauer et al., 2009). Further, an initial bout of FT stimulation can transform how a second bout of stimulation impacts spinal function, eliminating the deficit that each bout would induce on its own (Lee et al., 2015). As discussed above, this implies a form of savings across days. What is preserved, however, appears to be a coarse measure of regularity (soft timing). Hard timing, where a specific temporal interval is encoded and used in a flexible manner to modulate operant behavior may require brain systems. This difference in detail and flexibility parallels the limitations identified in other examples of spinal learning (Grau, 2014). While it is hardly surprising that brain mechanisms allow for more sophisticated forms of timing, it is also clear that spinal processes are sensitive to temporal relations. Further, independent of the type of clock at work, temporal relations impact spinal plasticity and for this reason, are relevant to rehabilitation after spinal injury.

Timing is a key component of learning and behavior, constraining the circumstances under which learning may occur and the performance of a conditioned response (Mauk and Buonomano, 2004). In describing these processes, researchers often implicitly adopt a kind of hourglass model. Our work suggests that timing within a relatively simple region of the nervous system depends upon an oscillatory process. Interestingly, analyses of the electrical activity associated with timing in a brain-dependent learning task also implicates neural oscillators (Caro-Martín et al., 2015). Indeed, it has been suggested that oscillatory devices may provide the foundation for coherent, well-timed, motor behavior (Llinás, 1988). From this view, what varies across levels of the neural axis is not the primitives used to generate behavior, but rather the flexibility with which these units can be coordinated, assembled, and regulated.

## Author Contributions

Conceived and designed the experiments: KHL, JWG. Performed the experiments: KHL, Y-JH. Analyzed the data: KHL, JWG. Contributed reagents/materials/analytic tools: JWG. Wrote the article: KHL, JWG.

## Conflict of Interest Statement

The authors declare that the research was conducted in the absence of any commercial or financial relationships that could be construed as a potential conflict of interest.

## References

[B1] Barbeau H. Rossignol S. (1987). Recovery of locomotion after chronic spinalization in the adult cat. Brain Res. 412, 84–95. 10.1016/0006-8993(87)91442-9 3607464

[B2] Barriere G. Leblond H. Provencher J. Rossignol S. (2008). Prominent role of the spinal central pattern generator in the recovery of locomotion after partial spinal cord injuries. J. Neurosci. 28, 3976–3987. 10.1523/JNEUROSCI.5692-07.2008 18400897 PMC6670475

[B4] Baumbauer K. M. Hoy K. C. Jr. Huie J. R. Hughes A. J. Woller S. A. Puga D. A.. (2008). Timing in the absence of supraspinal input I: variable, but not fixed, spaced stimulation of the sciatic nerve undermines spinally-mediated instrumental learning. Neuroscience 155, 1030–1047. 10.1016/j.neuroscience.2008.07.003 18674601 PMC2633135

[B5] Baumbauer K. M. Huie J. R. Hughes A. J. Grau J. W. (2009). Timing in the absence of supraspinal input II: regularly spaced stimulation induces a lasting alteration in spinal function that depends on the NMDA receptor, BDNF release and protein synthesis. J. Neurosci. 29, 14383–14393. 10.1523/JNEUROSCI.3583-09.2009 19923273 PMC2800823

[B7] Belanger M. Drew T. Provencher J. Rossignol S. (1996). A comparison of treadmill locomotion in adult cats before and after spinal transection. J. Neurophysiol. 76, 471–491. 8836238 10.1152/jn.1996.76.1.471

[B8] Bell-Pedersen D. Cassone V. M. Earnest D. J. Golden S. S. Hardin P. E. Thomas T. L.. (2005). Circadian rhythms from multiple oscillators: lessons from diverse organisms. Nat. Rev. Genet. 6, 544–556. 10.1038/nrg1633 15951747 PMC2735866

[B9] Boulos Z. Terman M. (1980). Food availability and daily biological rhythms. Neurosci. Biobehav. Rev. 4, 119–131. 10.1016/0149-7634(80)90010-x 6106914

[B11] Caro-Martín C. R. Leal-Campanario R. Sánchez-Campusano R. Delgado-Garciá J. M. Gruart A. (2015). A variable oscillator underlies the measurement of time intervals in rostral medial prefrontal cortex during classical eyeblink conditioning in rabbits. J. Neurosci. 35, 14809–14821. 10.1523/JNEUROSCI.2285-15.2015 26538651 PMC6605228

[B12] Cazalets J. R. Borde M. Clarac F. (1995). Localization and organization of the central pattern generator for hindlimb locomotion in newborn rat. J. Neurosci. 15, 4943–4951. 7623124 10.1523/JNEUROSCI.15-07-04943.1995 PMC6577873

[B13] Cha J. Heng C. Reinkensmeyer D. J. Roy R. R. Edgerton V. R. De Leon R. R. (2007). Locomotor ability in spinal rats is dependent on the amount of activity imposed on the hindlimbs during treadmill training. J. Neurotrauma 24, 1000–1012. 10.1089/neu.2006.0233 17600516

[B14] Crown E. D. Grau J. W. (2001). Preserving and restoring behavioral potential within the spinal cord using an instrumental training paradigm. J. Neurophysiol. 86, 845–855. 11495955 10.1152/jn.2001.86.2.845

[B15] Crown E. D. Joynes R. L. Ferguson A. R. Grau J. W. (2002). Instrumental learning within the spinal cord: IV. Induction and retention of the behavioral deficit observed after noncontingent shock. Behav. Neurosci. 116, 1032–1051. 10.1037/0735-7044.116.6.1032 12492302

[B16] de Leon R. Hodgson J. A. Roy R. R. Edgerton V. R. (1994). Extensor- and flexor- like modulation within motor pools of the rat hindlimb during treadmill locomotion and swimming. Brain Res. 654, 241–250. 10.1016/0006-8993(94)90485-5 7987674

[B17] de Leon R. D. Hodgson J. A. Roy R. R. Edgerton V. R. (1997). Locomotor capacity attributable to step training versus spontaneous recovery after spinalization in adult cats. J. Neurophysiol. 79, 1329–1340. 9497414 10.1152/jn.1998.79.3.1329

[B18] de Leon R. D. Hodgson J. A. Roy R. R. Edgerton V. R. (1998). Full weight-bearing hindlimb standing following stand training in the adult spinal cat. J. Neurophysiol. 80, 83–91. 9658030 10.1152/jn.1998.80.1.83

[B19] Dunlap J. C. (1999). Molecular bases for circadian clocks. Cell 96, 271–290. 10.1016/s0092-8674(00)80566-8 9988221

[B20] Duysens J. Van de Crommert H. W. (1998). Neural control of locomotion: the central pattern generator from cats to humans. Gait Posture 7, 131–141. 10.1016/s0966-6362(97)00042-8 10200383

[B21] Edgerton V. R. Tillakaratne N. J. Bigbee A. J. de Leon R. D. Roy R. R. (2004). Plasticity of the spinal neural circuitry after injury. Annu. Rev. Neurosci. 27, 145–167. 10.1146/annurev.neuro.27.070203.144308 15217329

[B22] Ferguson A. R. Crown E. D. Grau J. W. (2006). Nociceptive plasticity inhibits adaptive learning in the spinal cord. Neuroscience 141, 421–431. 10.1016/j.neuroscience.2006.03.029 16678969

[B23] Ferguson A. R. Huie J. R. Crown E. D. Baumbauer K. M. Hook M. A. Garraway S. M.. (2012). Maladaptive spinal plasticity opposes adaptive spinal learning after spinal cord injury. Front. Physiol. 3:399. 10.3389/fphys.2012.00399 23087647 PMC3468083

[B24] Frigon A. (2012). Central pattern generators of the mammalian spinal cord. Neuroscientist 18, 56–69. 10.1177/1073858410396101 21518815

[B26] Garraway S. M. Turtle J. D. Huie J. R. Lee K. H. Hook M. A. Woller S. A.. (2011). Intermittent noxious stimulation following spinal cord contusion injury impairs locomotor recovery and reduces spinal BDNF-TrkB signaling in adult rats. Neuroscience 199, 86–102. 10.1016/j.neuroscience.2011.10.007 22027236 PMC3237800

[B27] Garraway S. M. Woller S. A. Huie J. R. Hartman J. J. Hook M. A. Miranda R. C.. (2014). Peripheral noxious stimulation reduces withdrawal threshold to mechanical stimuli after spinal cord injury: role of tumor necrosis factor alpha and apoptosis. Pain 155, 2344–2359. 10.1016/j.pain.2014.08.034 25180012 PMC4253555

[B25] Gómez-Pinilla F. Huie J. R. Ying Z. Ferguson A. Crown E. D. Baumbauer K. M.. (2007). BDNF and learning: evidence that instrumental training promotes learning within the spinal cord by up-regulating BDNF expression. Neuroscience 148, 893–906. 10.1016/j.neuroscience.2007.05.051 17719180 PMC3225191

[B28] Gómez-Pinilla F. Ying Z. Roy R. R. Molteni R. Edgerton V. R. (2002). Voluntary exercise induces a BDNF-mediated mechanism that promotes neuroplasticity. J. Neurobiol. 88, 2187–2195. 10.1152/jn.00152.2002 12424260

[B29] Grau J. W. (2014). Learning from the spinal cord: how the study of spinal cord plasticity informs our view of learning. Neurobiol. Learn. Mem. 108, 155–171. 10.1016/j.nlm.2013.08.003 23973905 PMC3946174

[B30] Grau J. W. Barstow D. G. Joynes R. L. (1998). Instrumental learning within the spinal cord: I. Behavioral properties. Behav. Neurosci. 112, 1366–1386. 10.1037/0735-7044.112.6.1366 9926819

[B31] Grau J. W. Huie J. R. Garraway S. M. Hook M. A. Crown E. D. Baumbauer K. M.. (2012). Impact of behavioral control on the processing of nociceptive stimulation. Front. Physiol. 3, 1–21. 10.3389/fphys.2012.00262 22934018 PMC3429038

[B32] Grau J. W. Huie J. R. Lee K. H. Hoy K. C. Huan Y.-J. Turtle J. D.. (2014). Metaplasticity and behavior: how training and inflammation affect plastic potential within the spinal cord and recovery after injury. Front. Neural Circuits 8:100. 10.3389/fncir.2014.00100 25249941 PMC4157609

[B33] Grau J. W. Washburn S. N. Hook M. A. Ferguson A. R. Crown E. D. Garcia G.. (2004). Uncontrollable nociceptive stimu- lation undermines recovery after spinal cord injury. J. Neurotrauma 21, 1795–1817. 10.1089/neu.2004.21.1795 15684770

[B34] Grillner S. (1973). “Locomotion in the spinal cat,” in Control of Posture and Locomotion, eds Stein R. B. Pearson K. G. Smith R. S. Redford J. B. (New York, NY: Plenum Press), 515–535.

[B36] Grillner S. Wallén P. Brodin L. Lansner A. (1991). Neuronal network generating locomotor behavior in lamprey: circuitry, transmitters, membrane properties and simulation. Annu. Rev. Neurosci. 14, 169–199. 10.1146/annurev.neuro.14.1.169 1674412

[B35] Grillner S. Zangger P. (1979). On the central generation of locomotion in the low spinal cat. Exp. Brain Res. 34, 241–261. 10.1007/bf00235671 421750

[B37] Guertin P. A. (2009). The mammalian central pattern generator for locomotion. Brain Res. Rev. 62, 45–56. 10.1016/j.brainresrev.2009.08.002 19720083

[B38] Herzog E. D. (2007). Neurons and networks in daily rhythms. Nat. Rev. Neurosci. 8, 790–802. 10.1038/nrn2215 17882255

[B39] Hook M. A. Huie J. R. Grau J. W. (2008). Peripheral inflammation undermines the plasticity of the isolated spinal cord. Behav. Neurosci. 122, 233–249. 10.1037/0735-7044.122.1.233 18298266 PMC2665167

[B40] Huie J. R. Baumbauer K. M. Lee K. H. Beattie M. S. Bresnahan J. C. Ferguson A. R.. (2012a). Glial tumor necrosis factor alpha (TNFα) Generates metaplastic inhibition of spinal learning. PLoS One 7:e39751. 10.1371/journal.pone.0039751 22745823 PMC3379985

[B41] Huie J. R. Garraway S. M. Baumbauer K. M. Hoy K. C. Jr. Beas B. S. Montgomery K. S.. (2012b). Brain-derived neurotrophic factor (BDNF) promotes adaptive plasticity within the spinal cord and mediates the beneficial effects of controllable stimulation. Neuroscience 200, 74–90. 10.1016/j.neuroscience.2011.10.028 22056599 PMC3249495

[B42] Hultborn H. Nielsen J. B. (2007). Spinal control of locomotion - from cat to man. Acta Physiol (Oxf.) 189, 111–121. 10.1111/j.1748-1716.2006.01651.x 17250563

[B43] Hutchinson K. J. Gómez-Pinilla F. Crowe M. J. Ying Z. Basso D. M. (2004). Three exercise paradigms differentially improve sensory recovery after spinal cord contusion in rats. Brain 127, 1403–1414. 10.1093/brain/awh160 15069022

[B430] Ivry R. B. Schlerf J. E. (2008). Dedicated and intrinsic models of time perception. Trends Cogn. Sci. 12, 273–280. 10.1016/j.tics.2008.04.002 18539519 PMC4335014

[B44] Ivry R. B. Spencer R. M. (2004). The neural representation of time. Curr. Opin. Neurobiol. 14, 225–232. 10.1016/j.conb.2004.03.013 15082329

[B440] Joynes R. L. Ferguson A. R. Crown E. D. Patton B. C. Grau J. W. (2003). Instrumental learning within the spinal cord: V. Evidence the behavioral deficit observed after noncontingent nociceptive stimulation reflects an intraspinal modification. Behav. Brain Res. 141, 159–170. 10.1016/S0166-4328(02)00372-8 12742252

[B46] Joynes R. L. Janjua K. R. Grau J. W. (2004). Instrumental learning within the spinal cord: VI. Disruption of learning by the NMDA antagonist APV. Behav. Brain Res. 154, 431–438. 10.1016/j.bbr.2004.03.030 15313031

[B47] Karmarkar U. R. Buonomano D. V. (2007). Timing in the absence of clocks: encoding time in neural network states. Neuron 53, 427-438. 10.1016/j.neuron.2007.01.006 17270738 PMC1857310

[B48] Kiehn O. (2006). Locomotor circuits in the mammalian spinal cord. Ann. Rev. Neurosci. 29, 279–306. 10.1146/annurev.neuro.29.051605.112910 16776587

[B49] Kiehn O. Kjaerulff O. (1998). Distribution of central pattern generators for rhythmic motor outputs in the spinal cord of limbed vertebrates. Ann. N Y Acad. Sci. 860, 110–129. 10.1111/j.1749-6632.1998.tb09043.x 9928306

[B50] Lee K. H. Turtle J. D. Huang Y.-J. Strain M. M. Baumbauer K. M. Grau J. W. (2015). Learning about time within the spinal cord I: when does regularity matter and what is encoded? Front. Behav. Neurosci. 9:274. 10.3389/fnbeh.2015.00274 26539090 PMC4612497

[B51] Liu G. T. Crown E. D. Miranda R. C. Grau J. W. (2005). Instrumental learning within the rat spinal cord: localization of the essential neural circuit. Behav. Neurosci. 119, 538–547. 10.1037/0735-7044.119.2.538 15839800

[B52] Llinás R. R. (1988). The intrinsic electrophysiological properties of mammalian neurons: insights into central nervous system function. Science 242, 1654–1664. 10.1126/science.3059497 3059497

[B54] Magnuson D. Lovett R. Coffee C. Gray R. Han Y. Zhang P.. (2005). Functional consequences of lumbar spinal cord contusion injuries in the adult rat. J. Neurotrauma 22, 529–543. 10.1089/neu.2005.22.529 15892599

[B53] Magnuson D. S. Trinder T. C. Zhang Y. P. Burke D. Morassutti D. J. Shields C. B. (1999). Comparing deficits following excitotoxic and contusion injuries in the thoracic and lumbar spinal cord of the adult rat. Exp. Neurol. 156, 191–204. 10.1006/exnr.1999.7016 10192790

[B55] Maier S. F. Seligman M. E. P. (1976). Learned helplessness: theory and evidence. J. Exp. Psychol. Gen. 105, 3–46. 10.1037/0096-3445.105.1.3

[B56] Marder E. Bucher D. (2001). Central pattern generators and the control of rhythmic movements. Curr. Biol. 11, R986–R996. 10.1016/s0960-9822(01)00581-4 11728329

[B57] Mauk M. D. Buonomano D. V. (2004). The neural basis of temporal processing. Annu. Rev. Neurosci. 27, 307–340. 10.1146/annurev.neuro.27.070203.144247 15217335

[B59] Rossignol S. Barriere G. Alluin O. Frigon A. (2009). Re-expression of locomotor function after partial spinal cord injury. Physiology. (Bethesda) 24, 127–139. 10.1152/physiol.00042.2008 19364915

[B60] Rossignol S. Chau C. Brustein E. Belanger M. Barbeau H. Drew T. (1996). Locomotor capacities after complete and partial lesions of the spinal cord. Acta Neurobiol. Exp. (Wars) 56, 449–463. 8787206 10.55782/ane-1996-1148

[B58] Rossignol S. Frigon A. (2011). Recovery of locomotion after spinal cord injury: some facts and mechanisms. Ann. Rev. Neurosci. 34, 413–440. 10.1146/annurev-neuro-061010-113746 21469957

[B61] Roy R. R. Hutchison D. L. Pierotti D. J. Hodgson J. A. Edgerton V. R. (1991). EMG patterns of rat ankle extensors and flexors during treadmill locomotion and swimming. J. Appl. Physiol. (1985) 70, 2522–2529. 1885445 10.1152/jappl.1991.70.6.2522

[B62] Van de Crommert H. W. Mulder H. W. Duysens J. (1998). Neural control of locomotion: sensory control of the central pattern generator and its relation to treadmill training. Gait Posture 7, 251–263. 10.1016/s0966-6362(98)00010-1 10200392

[B63] van den Brand R. Heutschi J. Barraud Q. DiGiovanna J. Bartholdi K. Huerlimann M.. (2012). Restoring voluntary control of locomotion after paralyzing spinal cord injury. Science 336, 1182–1185. 10.1126/science.1217416 22654062

